# Integrated Health Care Barcelona Esquerra (Ais-Be): A Global View of Organisational Development, Re-Engineering of Processes and Improvement of the Information Systems. The Role of the Tertiary University Hospital in the Transformation

**DOI:** 10.5334/ijic.2476

**Published:** 2016-05-23

**Authors:** David Font, Joan Escarrabill, Mónica Gómez, Rafael Ruiz, Belén Enfedaque, Xavier Altimiras

**Affiliations:** Strategy and Planning Manager, Hospital Clínic, Barcelona 08036, Spain; Chronic Care Programme Manager. Hospital Clínic – AIS-BE, Spain; Director Master Plan for Respiratory Diseases (PDMAR) & Home Respiratory Therapies Observatory (Obs TRD) (Ministry of Health), Spain; REDISSEC (Research Network for Health Services in Chronic Disease), Spain; Coordinator of the AISBE Technical office, Spain; Eixample Primary Care Consortium (CAPSE) Manager, Spain; Primary Care Manager (SAP Esquerra – Catalan Health Institute), Spain; Integrated Health Area “Barcelona Esquerra” (AIS-BE) Manager. Barcelona Health Consortium (CatSalut), Spain; Standing Committee of AIS-BE, Spain

**Keywords:** integrated healthcare, clinical management, re-engineering of processes, shared knowledge and information

## Abstract

The Integrated Health Area “Barcelona Esquerra” (*Área Integral de Salud de Barcelona Esquerra* – AIS-BE), which covers a population of 524,000 residents in Barcelona city, is running a project to improve healthcare quality and efficiency based on co-ordination between the different suppliers in its area through the participation of their professionals. Endowed with an Organisational Model that seeks decision-taking that starts out from clinical knowledge and from Information Systems tools that facilitate this co-ordination (an interoperability platform and a website) it presents important results in its structured programmes that have been implemented such as the Reorganisation of Emergency Care, Screening for Colorectal Cancer, the Onset of type 2 Diabetes Mellitus, Teledermatology and the Development of Cross-sectional Healthcare Policies for Care in Chronicity.

## Introduction: Starting point and general goals

The fragmentation of healthcare, centred more on episodes than on processes, and the difficulties of co-ordination that this focus generates, are one of the causes that affect the quality of care [1]. The public expect safe health organisations that focus on people’s needs and are reliable. In order to achieve these goals the level of co-ordination of the health organisations may vary greatly [2,3]. So people talk of “integrated care”, “healthcare continuity”, “managed clinical networks”, “organised service delivery” and “integrated care organisations” among others [4,5,6], as key elements in the diverse models for integrating health services.

The purpose of this work is to describe the process of integration of healthcare in an urban area of the city of Barcelona based on four pillars: organisational development and re-engineering processes, improvements to information systems, systematic involvement of professional knowledge, and alignment of the management teams.

Healthcare in Barcelona is provided in the framework of the public health system based on the model of the National Health Service (universal cover, financed from taxation and free at the point of use). The organisation is structured in four integrated health areas, one of which is the Integrated Health Area of Barcelona Esquerra (*Área Integral de Salud de Barcelona Esquerra* – AIS-BE), the territory referred to in this study.

**Figure 1:**
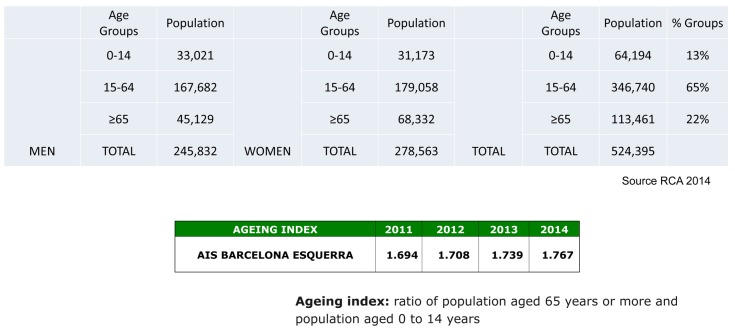
Population of the AIS Barcelona Esquerra (–).

The population covered by the AIS-BE is 524,000 residents, representing 35% of the population of Barcelona City and 7% of Catalonia. Figure 1 describes the characteristics of the population. Ageing was found to be above average for the population of Catalonia as a whole: 22% of people older than 65 yrs (19.8% of them older than 85 yrs). In Catalonia 17.3% of people are older than 65 yrs (17.3% of them older than 85 yrs).

Table 1 describes the various health services suppliers that serve the population financed by the National Health Service (in our case, the Catalan Health Service (CHS)). The coverage of social needs and the provision of services is the responsibility of the City. Medical facilities have social workers that perform social diagnosis and liaison tasks.

**Table 1:** Main healthcare suppliers in the AIS Barcelona Esquerra.

**Primary Care**

Institut Català de la Salut: 13 teams CAPSE: 3 teams EAP Poble Sec: 1 team EAP Sarrià-Vallplasa: 2 teams
**Specialty Care**

Hospital Clínic Hospital Plató Hospital Sant Joan de Déu Hospital Sagrat Cor
**Mental Health and Addictions**

Hospital Clínic Hospital Sant Joan de Déu SSM Hospital Sant Pere Claver Associació Centre Higiene Mental Les Corts Agència Salut Pública de Barcelona
**Social Health Care**

Parc Sanitari Pere Virgili Centre BlauClínic Clínica Sant Antoni de Barcelona Fundació Sociosanitària Barcelona


33 Community Pharmacies 4 Rehabilitation Centres Emergency Services (*Servei d’Emergències Mèdiques de Catalunya* – SEM)

In 2005 the CHS asked the Hospital Clínic to integrate all specialists working in isolation in the community in the hospital services. A diagnosis was made of the starting situation by Hospital Clínic and CHS and important dysfunctions were identified together with a great potential for improvement in co-ordination between the different suppliers to AIS-BE, notably:

- Variety among the suppliers, coexisting public bodies and private institutions with public funding, varying strategies and cultures and different information systems.
- Lack of a shared definition of goals and of a territory-based finance system.
- Incomplete definition of the role of each supplier, in caring for the basic disease and also in medium-high complexity.
- Potential for improvement in the scheduled processes, especially with the existence of specialists who work in the community without links to the hospitals in the area and with little ability for resolution, and co-ordination of the scheduled processes with emergencies. In particular, a great concentration of urgent activity in the tertiary hospital, the Hospital Clínic de Barcelona, with no information about previous scheduled activities carried out by the different institutions of the AIS-BE.
- Lack of knowledge among the various bodies and great resistance to change and fear of losing.

In this context, the main goals were to emphasise the priority in the integration of care pathways, not just in the integration of physicians to the hospital, to work to blur the boundaries between hospital and primary care, and to improve population health-related outcomes.

At the same time, the Hospital Clínic analysed the impact of chronic disease [7,8] on the centre itself and the problems connected with therapeutic adherence [9].

Starting from this initial diagnosis a project was set up to improve healthcare quality and public accessibility, based on co-ordination between the various suppliers and their professionals, seeking the optimal use of each care level in terms of efficiency. For the development of the project a follow-up body was set up with differentiated working groups to define the organisational structure, the information systems requirements and the care processes.

## Description of the care practice

The organisation of the AIS-BE is described in Table 2. The key element in the organisation of the AIS-BE is the existence of the Operational Committees (OC). The OCs were set according to the priorities defined in the Catalan Health Plan [10], of projects prioritised by the management structures of the AIS-BE or of needs identified by professionals themselves.

**Table 2:** Organisation of the AIS Barcelona Esquerra (AISBE).

Integrated Healthcare Committee Barcelona Esquerra (CAISBE)	Representation of the first management level of all suppliers involved. 1–2 meetings a year Monitoring the Strategic Plan and the main lines of work.
Standing Committee (SC)	Integrated Health Area “Barcelona Esquerra” (AIS-BE) Manager. Barcelona Health Consortium (CatSalut), Primary Care Manager of the Catalan Health Institute, CAPSE Manager, representative of each Hospital (Clínic, Plató, Sagrat Cor), Head of the Technical Office. Fortnightly meetings. Supervising execution of the plans and of the development of the work lines.
Technical Office (TO)	Staff of the Standing Committee comprising 3 professionals and support for professionals from the institutions Co-ordination and methodological support for the different Committees
Operational Committees (OC)	Consisting basically of medical and nursing personnel of the institutions. With a Co-ordinator for each Committee who reports to the TI and the SC. Methodological support for the TO. Proposals for improvements to organisation and processes, organising the role of each Hospital in relation with the Primary Care Teams both for the basic pathology and for tertiary care.

According to the four pillars strategy the organisational development and re-engineering process was defined around the priorities in patient care related to the specialities that the hospitals had to integrate. Some experiences are described in the next section.

In this process it was crucial to define the role of a teaching, high-tech and research focused hospital (Hospital Clínic) in an integrated care network. The challenge for Hospital Clinic was to work simultaneously as a dual hospital: high-tech (for the entire population of the AISBE and in some cases as a national reference centre) and as a community hospital (for 300,000 inhabitants). The framework of “dual hospital” was clear for the managerial structure but an iterative strategy was mandatory to progressively involve all front line clinicians.

**Figure 2:**
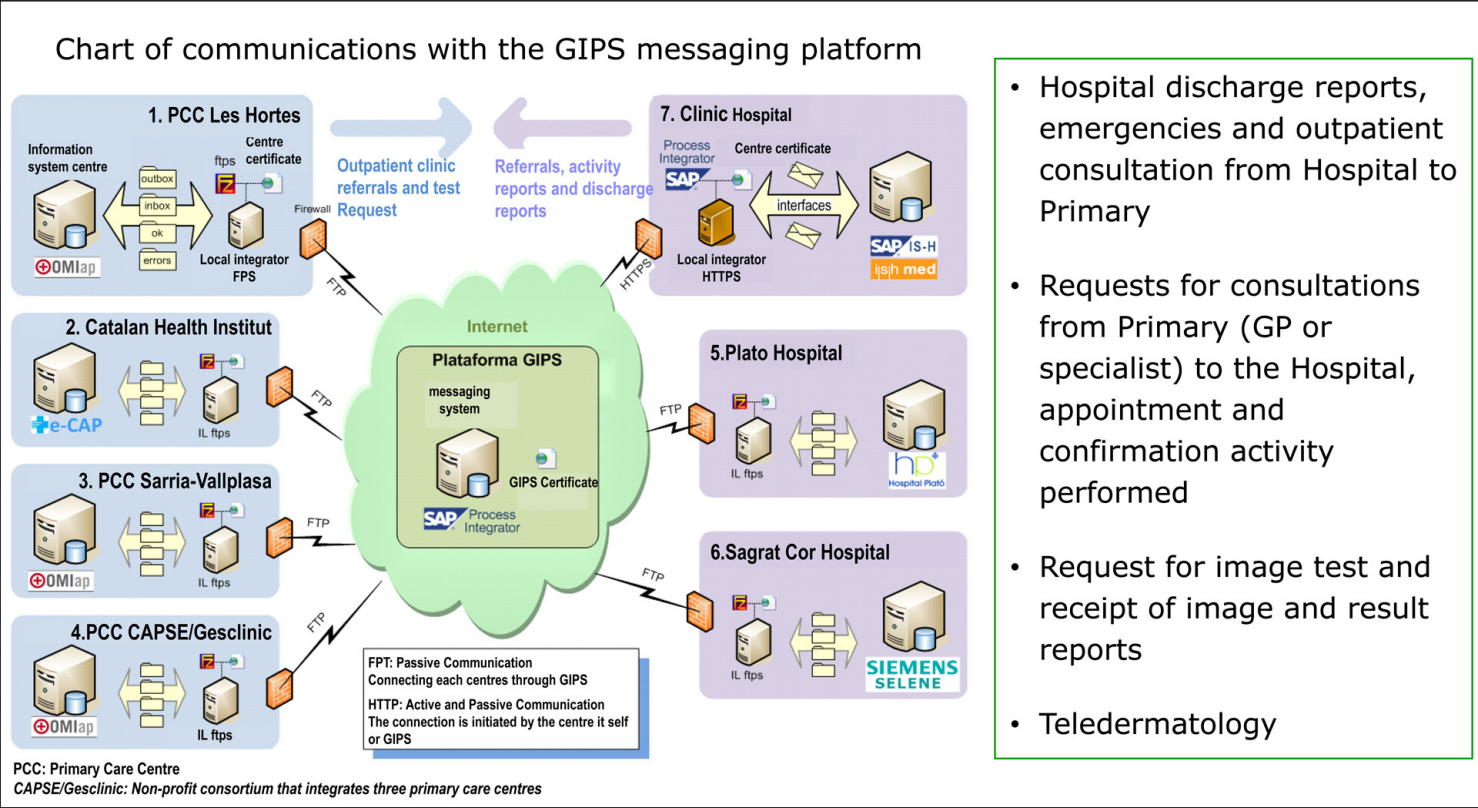
Interoperability platform and communication between the Information Systems of the suppliers of the AIS Barcelona Esquerr.

A platform has been implemented at the Information Systems level for interoperability and communication between suppliers, based on sending messages whose information is integrated in the suppliers’ differing information systems (Figure 2), with the following features:

- Reports of hospital admissions, emergencies and outside consultations of the Hospital that can be viewed immediately from Primary Care
- Appointment requests from Primary Care (GP or specialist) at the Hospital, with appointment and confirmation of the activity performed
- Request for image test and receipt of the image and of the report of results
- Telecare projects, e.g. teledermatology.

The AIS-BE has an intranet (www.ais-bcn.cat) to facilitate the collaborative work of the professionals in the territory, and its contents are the outcome of the work of the Operational Committees. The part of the information that is general to AIS-BE and the materials oriented to patients and the public have no restrictions on access.

The involvement of professional knowledge was considered crucial from the outset. The AIS-BE has more than 25 Operational Committees with the participation of more than 400 medical and nursing professionals from the different institutions and care levels. Table 3 shows the current OCs. Table 4 summarises, as an example, the activities of the Endocrinology operational committee.

**Table 3:** Operational Committees of the AIS Barcelona Esquerra.

Mental Health and Addictions	Locomotor Apparatus
Chronic Patient Care	Pain Clínic
Emergencies	Oncology and Haematology
Cardiology	Breast Cancer
General Surgery	Palliative Care
Vascular Surgery	Prevention and Community Health
Dermatology	Epidemiological Surveillance
Digestive	Sexually Transmitted Infections
Pneumology	Tropical Medicinal
Allergy	Ulcers
Neurology	Pharmacy
Endocrinology	Accessibility
Ophthalmology	Information Systems

**Table 4:** Main activities of the Endocrinology Operational Committee.

Definition of the role of primary care and the hospital by means of clinical pathways of the principal diseases. Organisation of a single Day Hospital facility to deal with the endocrinological emergencies of the whole territory. Implementation of case consultation sessions between GPS and endocrinologists. Identification of a reference nurse in diabetes in each Primary Care Team. Prioritising in drug prescription in a co-ordinated and integrated manner among the different care levels and suppliers. Implementation of a diagnostic and treatment programme for type II Diabetes in onset phase prioritising the group therapeutic education of the patients. Identification of uncontrolled diabetic patients in the territory and development of a co-ordinated intervention plan between the GP and the endocrinologist.

In practical terms there are more than 40 working groups (with physicians and nurses from hospital and primary care) that perform more than 80 activities mainly related to the definition of protocols and continuous education. More than 20 projects relate to improving the care through support of specialists to the community health services. Improving palliative care, intervention on poorly controlled diabetes patients, care for patients with allergic conditions, and renal diseases are examples of this type of intervention. Technology plays a key role in improving healthcare processes, as in the case of teledermatology (diagnosis of skin cancer or control skin ulcers) or diagnosis of diabetic retinopathy or macular oedema. In some cases, the work focuses on the reorganisation of the entire care process, as in the case of breast cancer or care for HIV patients. Five examples of these activities are described in detail in the next section.

**Figure 3:**
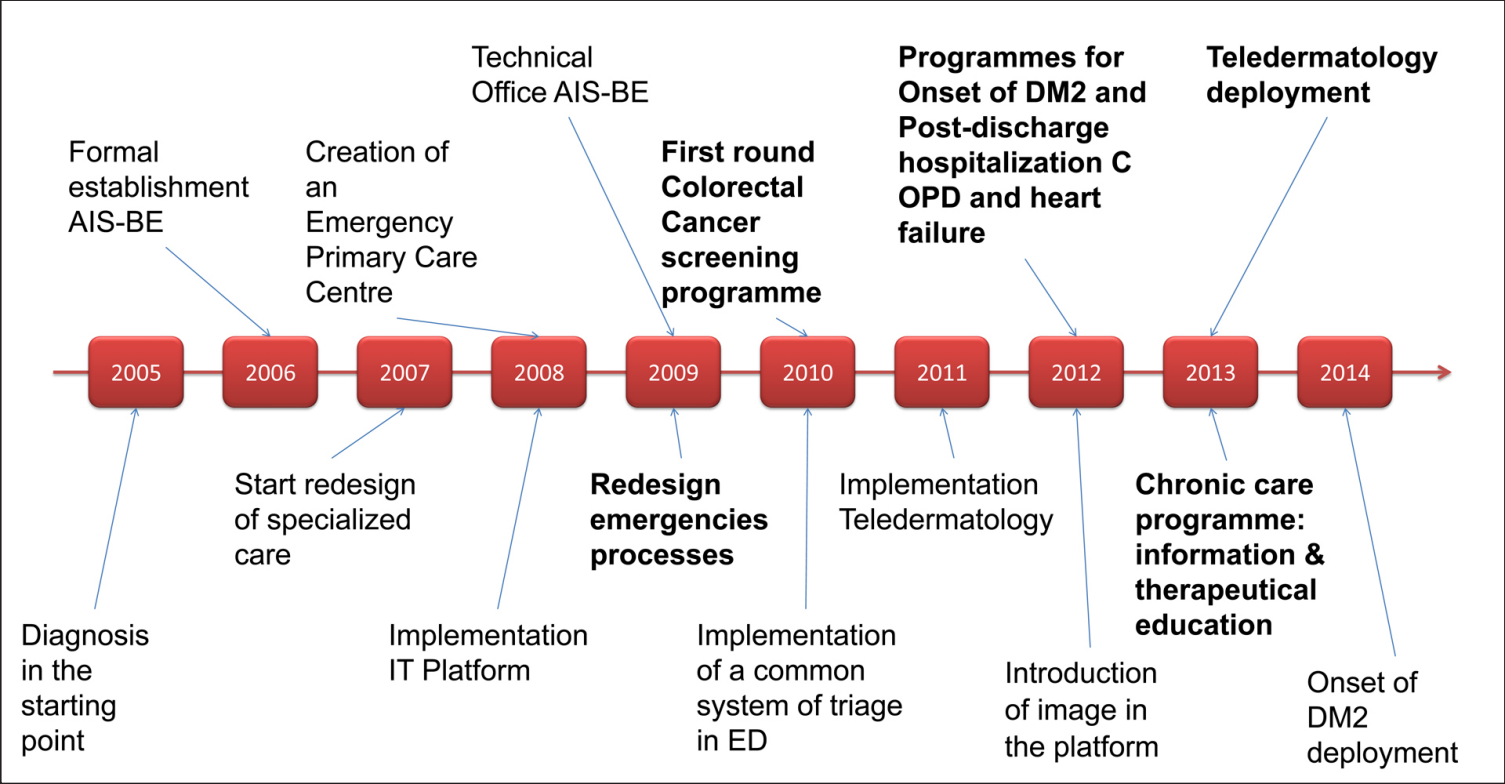
AIS-BE 2005–2016 10-year timeline key milestones.

Finally, the alignment of the management teams is mandatory. This is described to emphasise the importance of the approach from the clinical perspective and with the participation of healthcare professionals. For that reason a Standing Committee, including managers from primary care, hospitals and the Catalan Health Service was the first step to coordinate all activities and set priorities. Figure 3 shows a 10-year timeline of key milestones in the development of AIS-BE project.

## Five interventions that illustrate the development of the AIS-BE

As well as the general framework the transformation requires identification of the key elements in which, through the re-engineering of processes, the proposed changes are specified. Five interventions are described in the form of programmes that illustrate the development of the AIS-BE.

### Programme for Reorganisation of Urgent Care

#### Goal

To reorganise the healthcare devices and resources available for emergency care of the population aged >18 years in the AIS-BE, evolving from a highly centralised model in the A&E Service of the Hospital Clínic de Barcelona to a network care model adjusting the activity of each facility depending on the complexity, so as to free up A&E at the Hospital Clínic thereby improving waiting times.

#### Actions performed

Implementation of a homogeneous triage system in all the facilities in the territory (Andorran triage system, MAT) [11,12]; creation of an Emergency Primary Care Centre (CUAP Manso) for a resolution of less complex emergencies (MAT levels 4 and 5); reorganisation of emergency care [13] of the 3 hospitals responsible for care of patients aged >18 years and implementation of criteria for referral of complex pathology to the Hospital Clínic; evaluation of results [14].

#### Results

Table 5 describes the evolution of the activity during 2008–12, showing an overall reduction in consultations (19%) that is most noticeable in the Hospital Clínic (28.7%), especially in the less complex emergencies.

**Table 5:** Evolution of Emergencies consultations in the Barcelona Esquerra AIS: Activity and complexity.

A) Activity (Number of visits)							

	TOTAL 2008	TOTAL 2009	TOTAL 2010	TOTAL 2011	TOTAL 2012	TOTAL 2012 – 2008 Value %	

**Hospital Clínic**	**145,868**	**135,702**	**124,721**	**113,497**	**103,991**	**–41,877**	**–28.71%**
**H. Sagrat Cor**	**12,623**	**13,742**	**15,461**	**16,693**	**18,914**	**6,291**	**49.84%**
**H. Plató**	**7,953**	**9,207**	**9,847**	**12,299**	**12,964**	**5,011**	**63.01%**
**Total Hospitals**	**166,444**	**158,651**	**150,029**	**142,489**	**135,869**	**–30,575**	**–18.37%**

**CUAP Manso**	**43,067**	**59,177**	**60,090**	**63,634**	**53,867**	**10,800**	**25.08%**
**Global**	**209,511**	**217,828**	**210,119**	**206,123**	**189,726**	**–19,785**	**–9.44%**

B) Evolution of the complexity level of the emergencies dealt with in the Hospital Clínic			

	**2009**	**2010**	**2011**

**% level 4 and 5 emergencies (low complexity)**	**54%**	**38%**	**32%**

In the period September 2010 to April 2012, reduction in the waiting time at Emergencies at the Hospital Clínic, with 22 minutes decrease in level 4 (–26%), 13 minutes in level 5 (–18%) and 6 minutes in level 3 (–7%).

#### Planned actions

Evaluation of improvement actions in the care of chronic patients in the different emergencies facilities and analysis of the possibility of carrying out level 3 activities (intermediate complexity) by the CUAP Manso and also co-ordinating the direct admission to geriatric intermediate care units as a potential alternative to acute hospitalisation through emergency department for selected older patients [15,16].

### Colorectal Cancer screening programme

#### Goal

To implement a Colorectal Cancer Screening Programme based on faecal immunochemical testing [17] and the participation of the high street pharmacies as key agents in the management of the test.

#### Actions performed

Identification of the target population (50–69 years). involvement of the high street pharmacies, creation of a Screening Office consisting of specialist nursing staff who give information to patients if a test proves positive; definition of the role of primary care and of the hospitals in the territory in treatment and follow-up according to complexity; implementation of the first round (2 years); evaluation of results.

#### Results

The percentage of participation in the first round of screening (January 2010 to September 2012), was 44% and in 3.3% of the cases a pathological lesion was found.

#### Planned actions

To finish the implementation of the second round of screening, complete computerisation of the process through the interoperability platform and by assessing how the incidence and mortality from Colorectal Cancer evolve in Barcelona Esquerra. The Catalan Ministry of Health wants to expand the same model for the entire population of the region (7.5 million inhabitants).

### Programme for Onset of type 2 Diabetes Mellitus

#### Goal

To improve the control of type 2 diabetes from the moment of its onset through a group programme of structured therapeutic education [18].

#### Actions performed

Running the programme with design of the plan and material education and the evaluation indicators; implementation of improved record-keeping in the primary care IT system; training nursing staff for running the educational programme; implementation in three basic health areas (11,1847 residents); evaluation for extending to all of the territory.

#### Results

In 2013 191 patients took part in the programme. An increase was observed in the percentage of patients with HbA1c < 7 (from 50 to 82%), reduction in the percentage of smokers (from 22% to 17%), lowering of triglycerides (from 167 to 124 mg/dl) and increase in HDL (from 46 to 48 mg/dl). Also, the participants in the programme have reduced alcohol consumption and increased physical activity.

The frequency of attention in Primary Care was maintained and hospital attendance was reduced.

#### Planned actions

Extension of the Therapeutic Education Programme in the type 2 diabetes onset to all the population of Barcelona Esquerra. Starting from the identification of the uncontrolled diabetic patients in the territory, a co-ordinated intervention plan between the GP and the endocrinologist was developed.

### Teledermatology programme

#### Goal

To improve resolution times and decrease unnecessary consultations of dermatological processes in general and especially in the early diagnosis of cases with suspected skin cancer by means of a Teledermatology programme.

#### Actions performed

Design of the Programme with identification of the dermatological lesions suitable for diagnosis by Teledermatology; complete computerisation of the process through training GPs in the use of the dermatoscope [19] and of the camera from the designed protocol; implementation in 3 basic health areas (96,000 residents); evaluation for extension to all of the territory.

#### Results

In 2013 a total of 839 patients were managed through Teledermatology. The diagnosis was completed in 2.59 days (previously 6 months waiting) with identification of 50 malignant tumours (6%); 40% saving in attended consultations.

#### Planned actions

Extension of the Teledermatology Programme to all the population of Barcelona Esquerra.

### Development of Cross-sectional Healthcare Policies for Care in Chronicity

With the aim of consolidating the specific Chronicity Care programmes, in December 2012 cross-sectional policies were proposed, shared between primary care and the hospital. In the first phase three policies were proposed: a) information, therapeutic education and support in taking decisions, b) transitional care and c) geriatric care and pharmaceutical policy. These policies were developed from working groups made up of professionals from all the healthcare facilities involved.

In connection with the information and the therapeutic education an inventory was made of all the interventions performed in the Hospital Clínic to analyse the quality of the information, correct mistakes, avoid contradictions and identify gaps in information. The hospital has identified 325 information materials, 70 education activities and 37 structured programmes. Sixty-nine per cent of the information materials are considered to be acceptable (format, legibility, clarity). The hospital’s professionals call for more methodological training in connection with the structured therapeutic education programmes.

There are specific interventions in this field: the annual course on methodology of the educational programmes, the creation of a group including professionals and patients for support in designing information and education materials, and finally the inclusion of patient experience as a tool to identify opportunities for care improvement [20,21].

The Hospital Clínic has worked on Home Hospitalisation programmes for more than ten years [22,23]. In 2006 an Integrated Care Unit was set up [24] to respond both to acute care (with post-discharge care programmes for patients admitted for increased acuteness of chronic obstructive pulmonary disease or heart failure) and to patients in whom frailty is predominant. The aim of the transitional care policy is to extend the intervention field to include all kinds of transitions [25], both in the hospital (from the emergency service or the intensive care unit to the conventional unit) and in relation with the community (from the hospital to the home or to the intermediate care facilities).

Intervention in the pharmacological treatment is crucial from two perspectives: person-centred prescribing [26] and designing a form shared between the primary care and the hospital. This third policy has especial incidence in geriatric patients.

## Discussion

The integration of health service provision is a conceptual framework that is easy to share from a theoretical point of view but it raises problems when it comes to specifying actions and comparing the models and the results obtained. As Jiwani and Fleury [27] point out, most of the models for integration of services are hybrids and have been built up starting from an iterative process that receives the impact both of the general conditions (funding systems, health policies and social provisions) and of the local conditions (demography, types of supplier or available human resources).

There is no route map that will make integration a reality [28]. A process of change like that of AIS-BE has been based on a situation analysis as the start point and on the willingness of all the suppliers to share some basic items. Following the proposals of Evans et al. [29] the definition of AIS-BE’s integration strategy is designed from the perspective of the community (integration of all the suppliers who provide a service to the residents of the AIS-BE), to get results that increase value from the point of view of the patient [30] and through organisational changes without organisational mergers (at least in the first phase). The idea of four pillars (re-engineering of processes, information systems, systematic involvement of professional knowledge, and alignment of the management teams) is just a way to describe a complex process. The ultimate goal is the process of re-engineering to bring health care to the community. However, the first step to achieve this is the commitment of the management teams. It is clear that without a minimal improvement in the information system it will be very difficult to succeed. Nevertheless, the key element is the involvement of professionals with a bottom-up vision.

In the processes that do not start from scratch, and in those where care must continue to be provided at the same level while the organisational transformations are implemented, the method for tackling the problems tends to be opportunistic, according to the health care problems to be solved. When the vision has been shared among all the people involved it is necessary to deal with the relevant processes on which there is the chance to act, to bring about changes that are coherent with the vision and to advance progressively. This is the idea behind reorganisation of emergency care or intervention on aspects of specialist care that were generated by the need to reorganise the role of the specialists who were working in the community with no relationship with the hospital. One option was to integrate the specialists into the hospital and provide the service in a conventional manner. Another option was to integrate the specialists’ work with the primary care. The second option was chosen and an example is the care programme for the onset of diabetes. To implement this strategy the fourth pillar, alignment of the management teams, is essential.

It is not possible to separate organisational change from the implementation of IT systems (the second pillar). The diversity of organisations, each with its own information system, ruled out any possibility of a single clinical record for all the territory. The technological option was an interoperability platform that allows three basic functions: a) sharing information on-line (admission reports, images and laboratory results), b) progressively strengthening the care processes (referrals of patients according to pre-established criteria) and c) increasing and integrating the information available for evaluation. All of this is done without the need for far-reaching changes in the IT systems of each supplier. The interoperability platform is complemented with a website that gathers all the documents generated by the working groups comprising the professionals. A future scenario proposes a single outpatient information system oriented to health problems that would communicate through the interoperability platform with the intrahospital inpatient system, for the management of acute episodes with a high need for interventionist procedures. A tool from Business Datawarehouse at territory level should make it possible to extend the evaluation beyond research programmes and projects.

A crucial element in developing the process is sharing the values among all the people involved, especially among the professionals and the management teams. It is not unusual for the professionals (interested in offering quality care to specific patients) to have the feeling that the management teams (with responsibility for providing care to a population group) have different agendas. This is the “ethos gap” that Pendleton talks of [31]. The creation of the OCs (the third pillar) has been very important in order to emphasise the key role of clinicians. Without the clinical leadership of the health professionals, especially the physicians, the transformation processes are very difficult [32]. The transformation process also highlights the difficulties in disseminating the innovations to the organisation as a whole, beyond the group of most committed professionals [33].

The AIS-BE has no legal existence, no single management, and no model of financing for the territory, tools that are in theory more robust for developing integrated care; its results have been reached, therefore, with tools like clinical management, strategic planning and analysis of patient-centred processes that have made it possible to break down barriers between entities and care levels. That means that AISBE is closer to a professional network than a hierarchical organisation.

As for the strategy and the organisational model, the development of integrated care in the population area of Barcelona Esquerra is based on creating an organisation that favours professional participation; doctors and nurses nourish the project with improvements in the care programmes relying on the methodological support of the Technical Office. A second basic element of the AIS-BE is the strategic alignment of the different institutions especially thanks to the Standing Committee, from which the representatives of each institution have to fit the strategy and the needs of the AIS-BE into those of each entity; thinking in territorial terms and of the patient facilitates this. In this regard, it is important to point out the strategic positioning of the Hospital Clínic which defines its mission as dual, tertiary and community, and which sets out, in its Strategic Plan, that the programmes for prevalent and community pathologies must be deployed with the different institutions in the territory and outside the walls of the hospital. All in all, the Hospital Clínic opts for the strategy of breaking down the boundaries between the hospital and primary care, in the line of the “hospital without walls” [34] as proposed by the Royal College of Physicians.

The model and the tools developed in the AIS-BE must make it easier to tackle the great challenge facing the current health systems, care for chronicity and the transformation of a system that has to cure and care and in which the active participation of the educated patient is fundamental.

## Conclusion

The integration of the health services directed at a whole population by different suppliers is an iterative process. The challenge is to move from successful local pilots to feasible, high population impact, deployment strategies [35,36]. The minimum elements are management alignment, participation of the professionals and communication tools. We must mention the importance of the fact that a tertiary hospital has become aware of the importance of responding to the needs of the community without renouncing complexity (which is related to the tertiary status and that has to respond to needs that go beyond the immediate community).

With the working methodology consolidated, it has been shown that it is possible to reach a good level of integration, not only in specific programmes (diabetes, teledermatology) but also in more cross-sectional aspects of the care (as in the case of emergency care). From this point of view the immediate challenges concentrate on introducing improvements in the care process based on analysing the patient’s experience, dealing with shared clinical history for all the outpatient processes and working with an integrated global vision on key aspects like home care.
